# An Overview of NRF2-Activating Compounds Bearing α,β-Unsaturated Moiety and Their Antioxidant Effects

**DOI:** 10.3390/ijms23158466

**Published:** 2022-07-30

**Authors:** Melford Chuka Egbujor, Brigitta Buttari, Elisabetta Profumo, Pelin Telkoparan-Akillilar, Luciano Saso

**Affiliations:** 1 Department of Chemical Sciences, Rhema University Nigeria, Aba 453115, Abia State, Nigeria; 2 Department of Cardiovascular and Endocrine-Metabolic Diseases, and Aging, Italian National Institute of Health, 00161 Rome, Italy; brigitta.buttari@iss.it (B.B.); elisabetta.profumo@iss.it (E.P.); 3 Department of Medical Biology, Faculty of Medicine, Yuksek Ihtisas University, 06520 Ankara, Turkey; pelintelkoparan@gmail.com; 4 Department of Physiology and Pharmacology “Vittorio Erspamer”, Sapienza University of Rome, 00185 Rome, Italy; luciano.saso@uniroma1.it

**Keywords:** NRF2, KEAP1, α,β-unsaturated moiety, carbonyl, sulfonyl, sulfinyl, antioxidant, anti-inflammatory, Parkinson’s disease

## Abstract

The surge of scientific interest in the discovery of Nuclear Factor Erythroid 2 (NFE2)-Related Factor 2 (NRF2)-activating molecules underscores the importance of NRF2 as a therapeutic target especially for oxidative stress. The chemical reactivity and biological activities of several bioactive compounds have been linked to the presence of α,β-unsaturated structural systems. The α,β-unsaturated carbonyl, sulfonyl and sulfinyl functional groups are reportedly the major α,β-unsaturated moieties involved in the activation of the NRF2 signaling pathway. The carbonyl, sulfonyl and sulfinyl groups are generally electron-withdrawing groups, and the presence of the α,β-unsaturated structure qualifies them as suitable electrophiles for Michael addition reaction with nucleophilic thiols of cysteine residues within the proximal negative regulator of NRF2, Kelch-like ECH-associated protein 1 (KEAP1). The physicochemical property such as good lipophilicity of these moieties is also an advantage because it ensures solubility and membrane permeability required for the activation of the cytosolic NRF2/KEAP1 system. This review provides an overview of the reaction mechanism of α,β-unsaturated moiety-bearing compounds with the NRF2/KEAP1 complex, their pharmacological properties, structural activity-relationship and their effect on antioxidant and anti-inflammatory responses. As the first of its kind, this review article offers collective and comprehensive information on NRF2-activators containing α,β-unsaturated moiety with the aim of broadening their therapeutic prospects in a wide range of oxidative stress-related diseases.

## 1. Introduction

It is well established that molecules bearing α,β-unsaturated moiety constitute an essential class of electrophilic NRF2 modulators with therapeutic importance in a wide range of inflammatory and oxidative stress-mediated diseases such as Parkinson’s disease, Alzheimer’s disease, obesity, diabetes, cancer, osteoporosis, liver injury, multiple sclerosis and many others. Considering the crucial role of NRF2 in the modulation of inflammatory and oxidative processes, there is a lot of interest in the study of natural and synthetic substances capable of activating the NRF2/ KEAP1 pathway in order to design new therapeutic strategies to treat oxidative stress and inflammatory diseases. The structural peculiarity, natural abundance, facile synthetic procedures and diverse pharmacological activities of α,β-unsaturated moiety-bearing compounds including their ability to activate the NRF2/KEAP1 signaling pathway have made them important motifs of medicinal interest worthy of in-depth research. Compounds bearing α,β-unsaturated functionalities have been extensively studied [1,2,3]. Their ability to react with nucleophilic sites endows them with a multitude of biological functions including the nuclear factor erythroid 2 (NFE2)-related factor 2 (NRF2) signaling pathway activation [2,4,5,6]. Currently, NRF2, a transcription factor belonging to the cap ‘n’ collar subfamily, has become a subject of extensive research because it represents a crucial regulator of the cellular defense mechanisms against oxidative stress and xenobiotic.

Several α,β-unsaturated carbonyl, sulfonyl and sulfinyl compounds such as dimethyl fumarate (NCT00810836), curcumin (NCT01052025), chalcones and many vinyl organosulfur compounds are notable NRF2 activators [7,8,9,10] as shown in Table 1. Table 2 summarizes the α,β-Unsaturated moiety-bearing NRF2 activators in clinical trial or approved by FDA. Dimethyl fumarate (DMF) has been approved by FDA for multiple sclerosis, while the other ones are in their various stages of discovery and clinical trials [8,11,12]. Curcumin has been evaluated in clinical trials for diseases such as impaired glucose tolerance and insulin resistance (NCT01052025). However, it has not been approved for human use due to poor bioavailability and adverse effects [13]. Chalcone derivatives such as licochalcone A have been involved in clinical trials, it has been explored for human oral squamous cell carcinoma in combination with paclitaxel (NCT03292822). Several sulfonamides have been approved by FDA as antimicrobial agents, but vinyl sulfonamides are yet to be subjected to clinical trials [14]. Amongst the sesquiterpene lactones, parthenolide, a Tanacetum derived NRF2 activator, has vast therapeutic effect in inflammation and oxidative stress-mediated diseases, especially cancer. It is in clinical trial for cancer treatment (NCT00133341) (Table 2). Amongst anticancer drugs currently in clinical development, parthenolide is the most promising and the first to specifically delete HDAC1 proteins without affecting other class of 1/IIHDACs in several tissue and cancer cells [15]. Despite the antioxidant and anti-inflammatory activity of helenalin an Arnica Montana-derived NRF2 activator, it is not in clinical trial and its development as an anticancer agent has been retarded probably due to allergic effects and toxicity. Costunolide exhibits significant antioxidant and anti-inflammatory effects in cancer studies [16], but no clinical trial has been conducted yet.

The α,β-unsaturated carbonyl, sulfonyl and sulfinyl-bearing compounds that activate NRF2 accomplish the activation process via the same mechanism of action which involves electrophilic modification of NRF2 repressor KEAP1-cysteine residues [7,8]. The α,β-unsaturated carbonyl, sulfonyl and sulfinyl moieties play a significant role in the activation of the NRF2 signaling pathway via thiol-Michael addition reaction [9,17,18]. They are lipophilic, and this property enables their easy absorption by cells and passage through the plasma membrane thereby facilitating the activation of the cytosolic NRF2-KEAP1 signaling pathway [19,20,21]. In this way, they elicit antioxidant and anti-inflammatory molecular processes [22,23]. In addition to the pharmacokinetic and pharmacodynamic properties, studies have been conducted to explore lipophilicity as the central component of drug-like properties of α,β-unsaturated structure-bearing NRF2 activators/KEAP1 inhibitors, considering its role in the permeation of the cytosol where NRF2 activation is accomplished [24]. Moreover, it is also reported that the pharmacodynamic and pharmacokinetic properties of these NRF2 activators depend on their lipophilicity [24]. Several α,β-unsaturated moiety-containing compounds exhibit the ability to partition between a lipophilic organic phase and a polar aqueous phase, a property known as lipophilicity (log P/D). Lipophilicity is the most important physicochemical property that accounts for solubility, membrane permeability, drug absorption and distribution [25]. Primarily, it is a structural property that influences the physicochemical and biochemical properties of α,β-unsaturated moieties. It is usually employed in the structural modification of compounds to improve certain properties [26]. The lipophilicity of α,β-unsaturated moieties gives them high permeation across membrane, improves their oral bioavailability and influences their absorption, distribution, metabolism and elimination (ADME) properties and potency [27,28]. Alrubaie et al. [29] reported that α,β-unsaturated carbonyls exhibit moderate to high lipophilicity (1.14–6.53 log *p* values indicating hydrophobicity properties), and electrophilicity >3.00972 eV (calculated using the electronic chemical potential and the chemical hardness). The lipophilicity of carbonyl-based compounds has been reported and the NRF2 activity of α,β-unsaturated moiety-bearing compounds have been studied in few neurodegenerative diseases [30,31,32]. The reactivity of α,β-unsaturated-bearing compounds accounts for their diverse pharmacological activities including the ability to activate the NRF2 signaling pathway and also scavenge free radicals, an additional antioxidant quality. The pharmacological profile of these compounds will be further discussed in Section 7. Similarly, the structure–activity relationship (SAR) analysis of α,β-unsaturated-bearing NRF2 activators provides insight into the NRF2/KEAP1 activity of these compounds and enables the modification of the chemical structure of the compounds for improved activity. The combination of two α,β-unsaturated moieties in a single molecular structure will possibly increase their capacity to activate the NRF2 signaling pathway due to synergistic action. The NRF2-based SAR of these compounds will be further discussed in Section 8.

Although significant advances have been made in the identification of individual NRF2 activators bearing α,β-unsaturated moiety, not much is known about the influence of their chemical structure on NRF2 activation, reaction mechanism and pharmacology. Moreover, there is no comprehensive information on their NRF2-mediated therapeutic potentials. Thus, the present review provides the role of α,β-unsaturated moieties in NRF2 activation, their molecular mechanisms in the light of electrophilic modification of KEAP1 cysteine residues, pharmacological profile, NRF2 structure–activity relationship and their therapeutic effects in oxidative stress-mediated diseases.

## 2. Biologic Effects of NRF2/KEAP1 Signaling Pathway

It is well established that α,β-unsaturated moiety-bearing compounds activate the NRF2/KEAP1 signaling pathway. NRF2/KEAP1 pathway can be described as the chief regulator of endogenous antioxidant and cytoprotective responses to oxidative stress and inflammation. NRF2 is a transcription factor consisting of 605 amino acids and containing seven functional domains named Neh1-Neh7. The Neh1 domain contains a cap ‘n’ collar basic-region leucine zipper (bZIP) domain which is responsible for the binding to DNA [33] and a nuclear localization signal (NLS) that is involved in the nuclear translocation of Nrf2 [34]. The N-terminal regulatory domain Neh2 contains seven lysine residues and two peptide binding motifs (ETGE and DLG) and determines the stability and ubiquitination of Nrf2 by its negative regulator Keap1 [35,36]. The Neh3, Neh4 and Neh5 domains mediate the interaction of Nrf2 with other coactivators [37,38], while the Neh6 domain has a negative regulatory role as it promotes Nrf2 ubiquitination by binding to a β-transducin repeat-containing protein (β-TrCP) [39]. The Neh7 domain is responsible for the binding of NRF2 to the retinoic X receptor (RXR) and inhibits the NRF2-ARE signaling pathway [40]. Under homeostatic conditions, NRF2 is constitutively ubiquitinated by Kelch-like ECH- associated protein 1 (KEAP1), an adaptor component of the Cul3 (Cullin 3)-based ubiquitin E3 ligase complex and undergoes degradation by the proteasome [41,42]. In pro-oxidant and pro-inflammatory conditions, the exposure to electrophiles or oxidants alters the structure of NRF2/KEAP1 complex, thus preventing NRF2 ubiquitination and creating a non-functional KEAP1 complex. As NRF2 is not released by KEAP1, it saturates all binding sites of KEAP1, allowing newly translated NRF2 to bypass KEAP1 and translocate to the nucleus [43,44,45]. Within the nucleus, NRF2 heterodimerizes with members of small musculoaponeurotic fibrosarcoma (sMAF) family of transcription factors and binds to a regulatory enhancer sequence termed Antioxidant Response Element (ARE), thus promoting the expression of antioxidant and detoxifying genes and down-modulating the production of pro-inflammatory mediators [46,47].

NRF2 also cooperates with the NF-κB signaling pathway to control the response to oxidative stress and inflammation and to preserve the physiological homeostasis of cells [48]. NF-kB is a complex of transcription factors that regulates the expression of genes involved in the activation of innate and adaptive immunity, inflammation and oxidative stress responses [48]. In physiological conditions, NF-kB is retained in its inactive form in the cytoplasm of the cells by the inhibitory proteins belonging to the IkB family. When the cells are exposed to specific stimuli such as proinflammatory cytokines and oxidative stress, IkB proteins are phosphorylated, and this leads to their ubiquitination and proteasomal degradation. Consequently, NF-κB translocates into the nucleus and induces the expression of its target genes [49]. NRF2 negatively controls the NF-κB signaling pathway as it decreases intracellular ROS levels and counteracts oxidative stress-mediated NF-κB activation [50]. Furthermore, NRF2 prevents IκB-α proteasomal degradation, thus inhibiting the nuclear translocation of NF-κB [51]. In this way, NRF2 contributes to inhibit inflammatory and oxidative processes. NRF2 controls oxidative stress also by other mechanisms. In particular, evidence exist demonstrating the crucial role of NRF2 in the regulation of mitochondrial activity [52]. Mitochondria have a pivotal role in ROS production, and experimental data obtained in mice demonstrated that NRF2 is associated with the outer mitochondrial membrane and protects mitochondria from oxidative insults [53]. Another way by which NRF2 controls oxidative stress and inflammation is by modulating the expression of the enzyme Heme oxygenase (HO)-1. NRF2 activation increases cellular HO-1 levels and promotes the expression of phase II enzymes, thus inhibiting the degradation of IκB-α [54]. Different in vitro and in vivo experiments demonstrated the fundamental role of the NRF2-mediated expression of HO-1 in the activation of anti-inflammatory pathways. In particular, HO-1 activation promotes the secretion of the anti-inflammatory cytokine IL-10 in M2 macrophages and is associated with the anti-inflammatory activity in diabetes-associated gastric pathology [55,56].

A large body of evidence indicates that α,β-unsaturated moiety-mediated activation of the NRF2/KEAP1 signaling pathway modulates metabolic processes. It is also well known that oxidative stress and inflammation are involved in many chronic pathological conditions, and NRF2 is considered an interesting and promising therapeutic target. Indeed, NRF2 regulates the expression of several antioxidant enzymes such as NAD(P)H Quinone Dehydrogenase 1 (NQO1) and heme oxygenase-1 (HO-1), which are involved in xenobiotic metabolism [57], in metabolism of carbohydrates [58], lipids and iron [59], and modulates anti-inflammatory responses [48]. In vitro and transgenic model systems, as well as clinical and epidemiological studies have implicated NRF2 activity on the activation of endogenous antioxidant and cytoprotective mechanisms in the prevention and treatment of oxidative stress and inflammation-mediated diseases, including neurodegenerative diseases, cardiovascular disorders, autoimmune diseases, and lung, liver and kidney chronic diseases [7,8,9,60,61]. A large body of data consider it a paradox that NRF2 inhibits tumor initiation and cancer metastasis via the elimination of ROS and carcinogens but becomes an accomplice in helping tumor cells to withstand high level of ROS and resist apoptosis which can be referred to as the reverse side of the NRF2/KEAP1 signaling pathway. However, studies conducted on myeloid-derived suppressor cells (MDSCs) demonstrated an antitumor activity of NRF2 linked to the significant reduction in ROS levels and tumor metastasis determined by the inhibition of IL-6 secretion in MDSCs [62,63].

The NRF2/KEAP1 signaling pathway serves as an essential defense pathway that protects pancreatic β-cells against physiological and pathological attacks. It attenuates oxidative damage via the repression of apoptosis and proliferation in diabetic mice [64]. The modulation of the NRF2/KEAP1 pathway improves insulin sensitivity in diabetes and obesity [65]. It is also a potential method of ameliorating oxidative damage that occurs in allogenic islet cell transplantation [66]. In a mouse model of diabetes, it has been demonstrated that KEAP1 knockout, by promoting NRF2 activation, improved insulin secretion and insulin resistance and resulted in the prevention of hyperglycemia [65]. Of interest, the improvement of insulin secretion has been associated with the inhibition of IL-1 and IL-1 receptor expression [67]. It is worth noting that α,β-unsaturated moiety-bearing modulators of NRF2/KEAP1 play important therapeutic roles in bone diseases. Some of the α,β-unsaturated carbonyl-based sesquiterpene, such as parthenolide control systematic peroxidation state, regulate bone homeostasis and attenuates osteoporosis probably through the induction of antioxidant and repair enzymes. The NRF2/KEAP1 pathway has been found to mitigate bone loss, decrease fracture risk and reduce the incidence of osteoporosis [68]. The involvement of this pathway in artherosclerotic resistance is a therapeutic map in coronary artery disease [69]. Taken together, the activation of the NRF2/KEAP1 signaling pathway induces the expression of antioxidant genes such as HO-1, NQO1, GPX1, TXN, PRDX1 and suppresses NF-ĸB-dependent proinflammatory genes such as iNOS and COX2. This implies that NRF2/KEAP1 pathway is an essential therapeutic target in a wide range of diseases in which inflammation and oxidative stress have been implicated such as Parkinson’s disease, Alzheimer’s disease, diabetes, osteoporosis, atherosclerosis, rheumatoid arthritis, septic shock and many others. Other therapeutic effects of α,β-unsaturated moiety-mediated activation of the NRF2/KEAP1 signaling pathway in diseases have been highlighted in Table 1.

## 3. Modulation of NRF2/KEAP1 Signaling Pathway by α,β-Unsaturated Moiety-Bearing Compounds

**Scheme 1:**
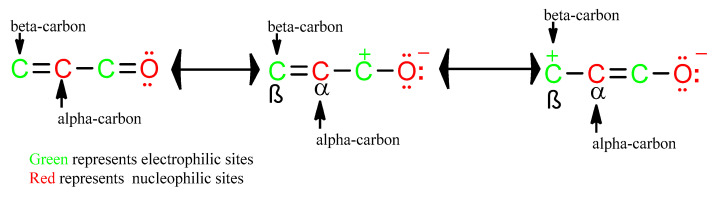
A resonance description of the transmission of electrophilic character to the beta-carbon of α,β-unsaturated carbonyl system (1).

**Scheme 2:**
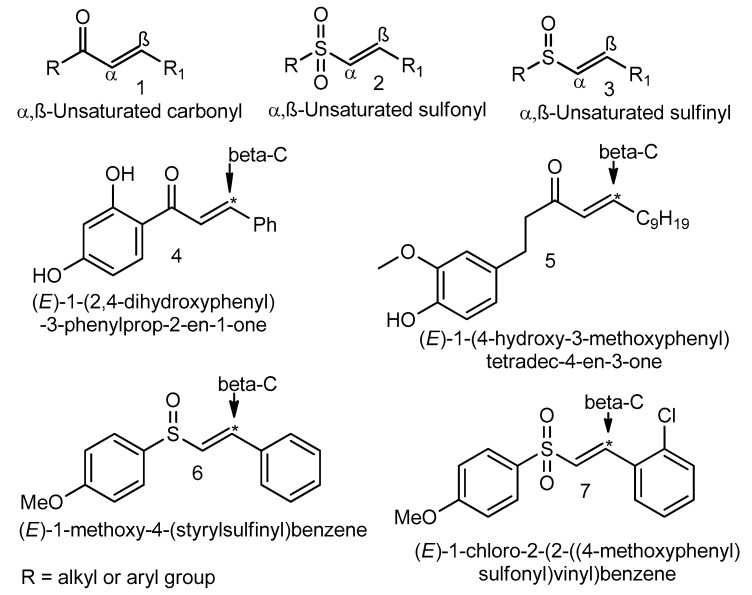
A schematic view of the electrophilic beta-carbon (indicated with asterisks) of α,β-unsaturated carbonyl (1), sulfonyl (2), sulfinyl (3) and some NRF2-activating compounds containing these α,β-unsaturated moieties (4–7). The asterisks represents the point at which thiols of cysteines are most likely to attack.

**Scheme 3:**
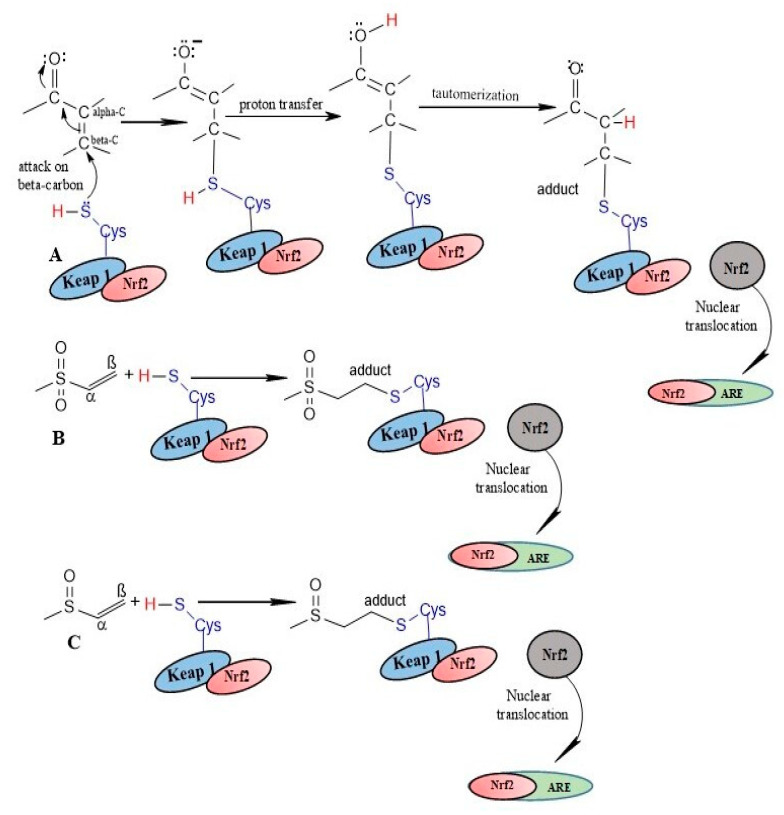
Reaction mechanisms of α,β-unsaturated (**A**) carbonyl, (**B**) sulfonyl and (**C**) sulfinyl moieties. The nucleophilic attack of the thiol of the KEAP1 cysteine residues on the β carbon of the carbonyl group is followed by 1,4-addition reaction in which the thiol bonds to carbon in position 1 and hydrogen bonds to oxygen in position 4. It undergoes tautomerization to form adducts which facilitates the nuclear translocation of NRF2 (**A**). The reaction of α,β-unsaturated sulfonyl (**B**) and α,β-Unsaturated sulfinyl (**C**) with thiols of the KEAP1 cysteine residue also enable NRF2 translocation.

Although carbonyl and sulfonyl groups are both electron-withdrawing, the sulfonyl group tends to exhibit more of an electron-withdrawing effect than the carbonyl group. It is therefore preferred to the carbonyl group as a leaving group in nucleophilic substitution reactions [70]. However, there is a more efficient delocalization with carbonyl groups than with sulfonyl groups [70]. The beta-carbon of the α,β-unsaturated carbonyl, sulfonyl and sulfinyl groups is the most reactive electrophilic atom of these groups [23,71]. There is electron deficiency at the beta-carbon of the α,β-unsaturated carbonyl, sulfonyl and sulfinyl groups due to the electron-attracting and delocalizing activity of these moieties, and this property accounts for their electrophilicity [17,72,73]. The electrophilic character is transmitted to the beta-carbon of the double bond following the conjugation of a double bond to a carbonyl, sulfonyl and sulfinyl group in α,β-unsaturated systems. This phenomenon favors 1,4-addition reaction [74]. The resonance description of the transmission of electrophilicity to the beta-carbon (Scheme 1) [74] confirms that the beta-carbon represents the electrophilic atom at which nucleophilic thiols of cysteines are most likely to attack. Thus, the beta-carbon of α,β-unsaturated carbonyl, sulfonyl, sulfinyl groups and that of NRF2 activators containing them (**4**–**7**) are indicated in Scheme 2. The nucleophilic attack of the α,β-unsaturated structural systems by thiols of the KEAP1 cysteine residues occurs via the reaction mechanism represented in Scheme 3.

The electrophilic modification of the cysteine residues of cytosolic proteins by α,β-unsaturated carbonyl, sulfonyl and sulfinyl groups has been found to affect transcriptional regulation of the NRF2 signaling pathway [4,7,23]. The NRF2 pathway is likely the most sensitive pathway for electrophilic thiol-modifying molecules due to the presence of several highly reactive cysteine residues in KEAP1 [75]. Under homeostatic conditions, there is a continuous degradation of NRF2 protein in the cytoplasm by a complex of E3 ubiquitin ligase containing the regulatory cysteine-rich KEAP1 protein [18,76]. However, under oxidative stress, electrophilic α,β-unsaturated carbonyl, sulfonyl and sulfinyl compounds modify Keap1 [9,71,77]. They react with some cysteine residues of KEAP1 to form adducts that create a non-functional KEAP1 complex, thus favoring the nuclear translocation of newly translated NRF2 and facilitating transcriptional induction of NRF2–dependent genes [78,79,80,81]. Many cysteines of KEAP1 are modified by different electrophiles [78,79,82,83,84,85]. KEAP1 is a cysteine-rich protein possessing 27 and 25 cysteine residues in the human and mouse proteins, respectively. This “cysteine-code” controls KEAP1 activity. Cysteines Cys-151, Cys-273 and Cys-288 [86,87] appear to be the most susceptible to electrophilic reaction [85,88]. Based on the functional necessity of these three cysteine residues in the maintenance of KEAP1 ability to inhibit NRF2 accumulation, chemical inducers of NRF2 were categorized into four classes in relation to the cysteine on which they act [85], namely, class I (Cys151preferring), class II (Cys288 preferring), class III (Cys151/Cys273/Cys288 collaboration preferring) and class IV (Cys151/Cys273/Cys288 independent). Other sensitive cysteines are Cys-226, Cys-434 and Cys-613. Thus, considering the distinct patterns of adduct formation for each chemical inducers of NRF2, the set of optimal acceptor thiols that are functional and convert KEAP1 from the active to the inactive state should be determined.

**Scheme 4:**
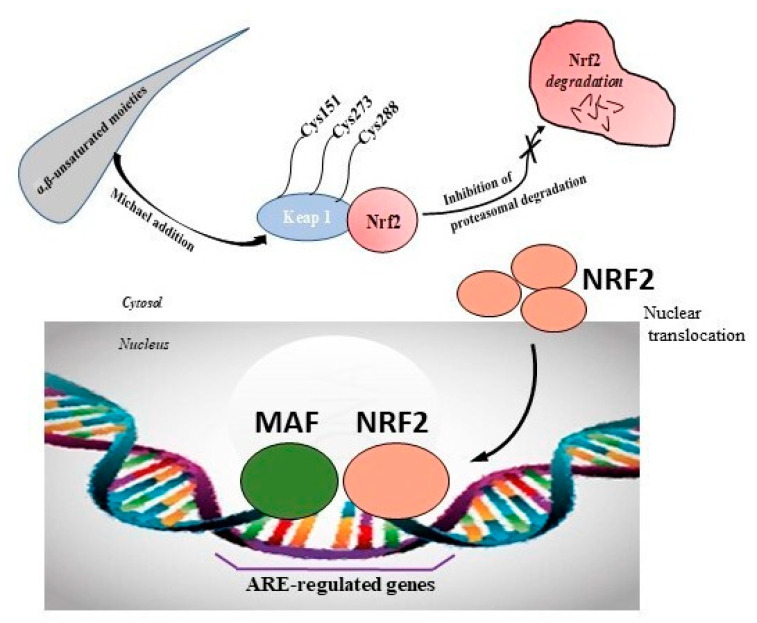
Mechanism of activation of KEAP1-NRF2-ARE pathway by α,β-unsaturated moieties. In pro-oxidant condition, the exposure to electrophilic α,β-unsaturated moieties alters the structure of NRF2/KEAP1 complex, thus inhibiting NRF2 ubiquitination and creating a non-functional KEAP1 complex. As NRF2 is not released by KEAP1, it saturates all binding sites of KEAP1, allowing newly translated NRF2 to bypass KEAP1 and translocate to the nucleus.

The NRF2 activation mechanism of α,β-unsaturated moieties is represented in Scheme 4. The α,β-unsaturated sulfonyl group (**2**) acts as a 2 donor and a Michael acceptor in addition reactions [89]. The stability of the α,β-unsaturated sulfonyl and sulfinyl systems needs to be understood. The equilibrium of these functionalities can be attributed to factors such as the interaction of the α,β-double bond with the d-orbitals of sulfur in addition to the inductive effects of the sulfonyl and sulfinyl groups. In the α,β-unsaturated sulfonyl and sulfinyl systems, the double bond stabilizes by interacting with sulfur’s d-orbitals. Inductive effects on the other hand, accounts for the electron withdrawing ability of the α,β-unsaturated sulfonyl and sulfinyl groups at equilibrium in the order sulfinyl < sulfonyl. The stability of the sulfonyl group, especially sulfones, has been linked to the strength of its carbon-sulfur bond. The observed minimal role of resonance effects and the major role of inductive effects suggest that the latter is very important in the stability of these systems. The α,β-unsaturated carbonyl systems are thermodynamically more favored than α,β-unsaturated sulfonyl and sulfinyl systems, while the α,β-unsaturated sulfonyl group is more stable than the α,β-unsaturated carbonyl system [90,91,92]. Sulfonyl functional group confers dienophilic activity to the double bond attached to it [93]. The double bond in α,β-unsaturated sulfonyl-containing compounds is activated by the sulfonyl group [94]. In parallel, Choi et al. [17] reported that the α,β-unsaturated sulfonyl system is a highly active Michael acceptor for NRF2 activation. The addition of hard nucleophiles to α,β-unsaturated sulfonyl system poses some difficulties due to metalation and conjugate additions occurring as competing reactions [95]. However, the addition of soft nucleophiles, especially thiols, to the α,β-unsaturated sulfonyl group via an addition reaction is an easy and effective process [96,97].

## 4. α,β-Unsaturated Carbonyls

α,β-Unsaturated carbonyl (**1**) compounds can be described as organic compounds with the general structure (O=CR)-C=C-R, in which carbonyl functional group is conjugated with an alkene [98]. For example, enones and enals exhibit vinylogues reactivity pattern which makes them prone to attack by nucleophiles at the beta-carbon [98]. In α,β-unsaturated carbonyl-based compound, one C-C bond separates the C=C and C=O bonds. The α,β-unsaturated carbonyl functionality is the most reactive substructure of synthetic and natural molecules [99,100]. The reactivity of this group explains its various pharmacological activities [100]. α,β-unsaturated carbonyls scavenge free radicals via covalent ligand binding to target proteins. They exhibit significant antioxidant and anti-inflammatory activities by thiol trapping [100,101,102]. Data have shown that α,β-unsaturated carbonyls react with a wide range of Cys-containing amino acids, proteins and peptides [73,103]. They exhibit different molecular actions due to localization and concentration in the different targeting of certain Cysteine residues on specific proteins. Experiments performed utilizing KEAP1 mutants have demonstrated that Cys-151, Cys-273 and Cys-288 are most sensitive to electrophilic reactions with the α,β-unsaturated carbonyl group and are essential for KEAP1 to inhibit Nrf2 activity [104,105,106]. Although few α,β-unsaturated carbonyl compounds such as acrolein and its derivatives are toxic, a good number of them induce adaptive or protective responses, exhibit remarkable NRF2 activity and play important signaling functions [107,108,109,110]. Several NRF2 activators strongly depend on the presence of the α,β-unsaturated carbonyl moiety for efficacy. The α,β-unsaturated carbonyl functionality is responsible for the reactivity of several NRF2 activators, including flavones and flavonols, and when this structural feature is disrupted, the ability of these compounds to activate NRF2 is completely suppressed. Moreover, the α,β-unsaturated carbonyl group is required by polyphenols to play the role of antioxidant via NRF2 activation. Wu et al. [111] reported that α,β-unsaturated carbonyl compounds activate NRF2 pathway, and the loss of the α,β-unsaturated carbonyl moiety abrogates the NRF2 activation by these compounds. Molecules containing α-β unsaturated carbonyl groups have been shown to activate NRF2 in a reporter system and normal peripheral blood mononuclear cells [112]. In line with this, we highlighted the α,β-unsaturated carbonyl-based compounds that have the ability to significantly activate the NRF2 signaling pathway in Table 2. However, several α.β-unsaturated-bearing electrophilic NRF2 activators may have the risk of ‘off-target’ effect as a result of their complex molecular mechanism of action which may affect their clinical development [113].

### 4.1. Sesquiterpene Lactones

Sesquiterpene lactones are sesquiterpenoids with a lactone ring, commonly obtained from Asteraceae plant family. They are lipophilic solids that serve as a rich source of drugs because of their wide range of biological activities including antioxidant and anti-inflammatory properties [112,114,115]. Sesquiterpene lactones (Table 1) such as parthenolide (**8**), helenalin (**9**), alantolactone (**10**) and costunolide (**11**) have been found to significantly activate the NRF2/KEAP1 signaling pathway in different in vitro cell culture systems [116,117,118,119]. Experiments performed in rat neuronal cells demonstrated that treatment with sesquiterpene lactones promoted nuclear NRF2 translocation and ARE target genes expression, and that ARE activation was dependent on the number of α,β-unsaturated carbonyl groups present in each compound [117]. These observations strongly suggest that the bioactivities of sesquiterpene lactones, especially their ability to activate the NRF2 pathway, can be attributed to the presence of the α,β-unsaturated carbonyl unit [117,120].

#### 4.1.1. Parthenolide

Parthenolide (**8**) (Table 1) is an α,β-unsaturated carbonyl-containing sesquiterpene lactone, the most abundant and active electrophilic compound obtained from feverfew plant (*Tanacetum parthenium*) [121,122]. The α,β-unsaturated lactone is reported to be the reactive part of parthenolide, not the epoxide [110]. The α,β-unsaturated carbonyl group is responsible for the electrophilic nature of parthenolide (**8**), which accounts for its ability to undergo Michael addition reaction with biochemical nucleophiles, to covalently modify proteins, and to activate the NRF2 pathway [123,124]. Kim et al. [124] reported that the antioxidant and anti-adipogenic effects of parthenolide are associated with NRF2 activation. Parthenolide (**8**) inhibits the early stage of adipogenesis, reduces the production of intracellular reactive oxygen species (ROS) and increases the expression of heme oxygenase-1 (HO-1) and NADPH dehydrogenase 1(NQO1) via the activation of the NRF2/KEAP1 signaling pathway [124]. In a similar study, Kim and co-workers [125] attributed the anti-obese effects of parthenolide (**8**) to its ability to activate NRF2. They reported that parthenolide (**8**) suppresses adiposity-induced inflammatory responses, controls the dysregulation of adiponectin and resistin, upregulates HO-1 and promotes nuclear translocation of NRF2 in obesity and related diseases. In summary, parthenolide inhibits obesity and obesity-related inflammatory responses through the activation of the NRF2/Keap1 signaling pathway. Mao and Zhu [126] reported that parthenolide (**8**) increases the expression of NRF2, HO-1 and NQO1 in hydrogen peroxide-induced osteoblasts, thereby preventing apoptosis by the reduction in oxidative stress. Parthenolide (**8**) exhibits significant anti-tumor and anti-inflammatory activities, it inhibits inflammatory mediators and the expression of pro-inflammatory cytokines [127,128]. Additionally, the anticancer activities of parthenolide are linked to its NRF2 activity, in particular it increases the level of glutathione via the activation of the NRF2-ARE signaling pathway [129,130]. The antioxidant activity of parthenolide is dose-dependent, at low dose (<5 µM), it neutralizes hydrogen peroxide and protects against CD3-induced apoptosis in Jurkat T cells, while at high dose (10 µM) it induces oxidative stress [131]. Of note, in recent studies aimed at identifying new strategies to overcome chemoresistance and to increase the effectiveness of chemotherapy in cancer, parthenolide was found to suppress mammosphere formation and overexpression of NRF2 and its dependent genes in triple-negative breast cancer cell lines, thereby preventing resistance to doxorubicin and mitoxantrone based on ROS modulation [132,133]. It was also reported that parthenolide (**8**) activates NRF2 and it is selectively cytotoxic to chronic lymphocytic leukemia (CLL) [111].

#### 4.1.2. Helenalin

Helenalin (**9**) (Table 1) is a sesquiterpene lactone obtained from Arnica montana and Arnica chamissonis foliosa containing an α,β-unsaturated carbonyl group that accounts for its anti-inflammatory, antioxidant, anti-cancer and NRF2 activities [134,135,136,137]. Lin et al. [138] reported that helenalin (**9**) inhibits oxidative stress, enhances ethanol metabolism and therefore attenuates alcohol-induced hepatic fibrosis. Li et al. [137] demonstrated that helenalin (**9**) isolated from Centipede minima (the family Asteraceae) exhibits significant antioxidant activity and anti-inflammatory effects by inhibiting NF-ĸB activation. It ameliorates acute hepatic injury, alleviates hepatocyte apoptosis, restores mitochondrial function and inhibits hepatic inflammatory cytokines. Helenalin (**9**) also alleviates lipid peroxidation, reduces ROS and NO production, increases antioxidant enzyme activity and HO-1 activity via activation of the NRF2 signaling pathway [137].

#### 4.1.3. Alantolactone

Alantolactone (**10**) (Table 1) is a sesquiterpene lactone commonly obtained from lnula helenium *L*. It contains α,β-unsaturated carbonyl moiety. It exhibits anti-inflammatory, antioxidant, anticancer and antibacterial activities [139,140,141]. According to Liu et al. [142], alantolactone (**10**) increases the expression and nuclear translocation of NRF2. This implies that the ability of alantolactone (**10**) to promote apoptosis and suppress migration in human breast cancer cell line may depend on NRF2 signaling in addition to other pathways such as p38 and NF-ĸB. Soe et al. [143] reported that the induction of detoxifying enzymes by alantolactone (**10**) is mediated by NRF2. Alantolactone (**10**) enhances the activity of glutathione and increases the induction of phase II and antioxidant enzymes such as glutathione reductase, heme oxygenase-1 and γ-glutamylcysteine synthase via the NRF2-ARE signaling pathway. It increases the nuclear translocation and activation of NRF2 in murine hepatoma (Hepa1c1c7) cells [143]. In vitro experiments conducted on human bronchial epithelial Beas-2B and NHBE cells demonstrated that alantolactone is able to prevent cigarette smoke extract (CSE)-induced pro-inflammatory cytokine production, caspase-3 activation and the increased levels of the oxidative stress markers malondialdehyde, ROS and superoxide dismutase. The same study also demonstrated that alantolactone promotes NRF2 nuclear aggregation and HO-1 expression, thus suggesting that this compound inhibits CSE-induced inflammation, apoptosis and oxidative stress by promoting NRF2 activation [144].

#### 4.1.4. Costunolide

Costunolide (**11**) (Table 1) is a sesquiterpene lactone usually obtained from Inula helenium and Vladimiria souliel [145]. It has been extensively studied due to its numerous biological functions such as anti-inflammatory, antioxidant and neuroprotective activities [145,146]. Pae et al. [147] reported that costunolide (**11**) reduces inflammation by the up-regulation of HO-1 expression. Furthermore, costunolide (**11**) has been reported to improve the level of GSH in tissues and to ameliorate ethanol-induced gastric ulcer through its antioxidant anti-inflammatory activities [148,149]. Peng et al. [150] demonstrated that costunolide (**11**) prevents oxidative injuries and hinders apoptosis by promoting the nuclear translocation of NRF2, and up-regulating the expression of NRF2 downstream molecules in the neuron-like rat pheochromocytoma cell line (PC12). It upregulates antioxidant genes and reduces cellular ROS levels thus maintaining redox balance in PC12 cells. However, the knockdown of NRF2 reportedly abrogated the cytoprotective activity of costunolide (**11**), thus suggesting that its ability to promote neuroprotection is dependent on NRF2 pathway activation. In another study, costunolide (**11**) was found to induce HO-1 expression and NRF2 nuclear accumulation, to inhibit pro-inflammatory cytokines and to activate NRF2 in RAW 264.7 macrophages [147]. Similarly, Mao et al. [146] reported that costunolide (**11**) inhibits lipopolysaccharide and D-galactosamine-induced acute liver injury via NRF2 activation. It also down-regulates KEAP1 gene expression and up-regulates HO-1 and NQO1 gene expressions. Taken together, these results indicate that costunolide (**11**) exerts protective effects against acute liver injuries via its antioxidant activity by promoting the NRF2 signaling pathway.

### 4.2. Curcumin

Curcumin (**12**) (Table 1) is a phytochemical usually obtained from rhizomes of Curcuma longa that exhibits significant antioxidant and anti-inflammatory activities [151,152]. It contains an α,β-unsaturated carbonyl group that accounts for its neuroprotective effect via NRF2 activation. It has been found to promote the nuclear expression levels and biological effects of NRF2 through the interaction of the α,β-unsaturated carbonyl moiety with Cys151 in KEAP1 [153,154]. According to a recent report by Park and co-workers [155], curcumin (**12**) induces the expression of NRF2-dependent genes such as NQO1, GST-mu1 and HO-1 and increases the level of NRF2 protein in neuronal cells. The activation of NRF2 by curcumin (**12**) is reportedly accomplished via PKCα- mediated P62 phosphorylation at Ser351 [155]. Similarly, Ashrafizadeh et al. [156] reported that curcumin activates the NRF2 signaling pathway by inhibiting KEAP1, up-regulating the expression of NRF2 and its dependent genes and promoting nuclear translocation of NRF2. The pre-treatment with curcumin (**12**) prevents hemin-induced neuronal death by inducing NRF2 and antioxidant response in cultures of cerebellar neurons of rats [157]. Curcumin (**12**) also inhibits the upregulation of inflammatory signaling-mediated KEAP1 synthesis and reduces NRF2 degradation in HepG2 cells [158]. Furthermore, curcumin (**12**) hinders oxidative stress in human nasal fibroblasts that have been exposed to urban particulate matter via the activation of the NRF2/HO-1 signaling pathway [159]. Of note, although curcumin (**12**) has been found to alleviate oxidative stress, the co-administration of curcumin and vitamin E gives a better result [160]. Co-treatment with vitamin E and curcumin of hypo- and hyper- thyroid rats resulted more efficient in down-regulating oxidative stress evaluated as lipid peroxidation and glutathione levels, and in promoting activities and protein expression of antioxidant enzymes such as superoxide dismutase, catalase, glutathione peroxidase and glutathione reductase, when compared to individual treatment. In the same study, a modeled active portion of the protein NRF2 indicated its interaction with both vitamin E and curcumin. Furthermore, in silico experiments showed the interaction of curcumin and vitamin E complex with KEAP1, suggesting that the more effective attenuation of oxidative stress by the concomitant administration of these two antioxidants might be the result of NRF2/KEAP1 pathway modulation [160].

### 4.3. J-Series Cyclopentenone Prostaglandin

15-Deoxy-D-prostaglandin J_2_ (15d- PGJ2) (**13**) (Table 1) is a peroxisome proliferator-activated receptor γ ligand. It represents the J-series cyclopentenone prostaglandin and exerts cytoprotection via NRF2-mediated induction of antioxidant enzymes due to the presence of α,β-unsaturated carbonyl moiety [161,162]. Song et al. [163] corroborated the importance of the α,β-unsaturated carbonyl group in NRF2 activation by demonstrating that 9,10-dihydro-15d-PGJ2 (H_2_-15d-PGJ_2_), an analogue of 15d-PGJ2 that lacks α,β-unsaturated carbonyl moiety as a Michael acceptor, is not able to induce the NRF2 signaling pathway. 15d-PGJ2 (**13**) induces the up-regulation of multidrug resistance associated proteins through the activation of the NRF2-ARE signaling pathway [164]. It has been found to regulate the expression of NRF2-dependent genes and enzymes [36]. However, NADPH-dependent alkenal/one oxidoreductase reportedly attenuated the ability of 15d-PGJ2 (**13**) to affect NRF2-mediated induction of cytoprotective enzymes [164].

### 4.4. Chalcone and Its Derivatives

Chalcone and its derivatives (Table 1) exhibit significant antioxidant, anti-inflammatory and anticancer activities [165,166,167,168]. Their ability to activate the NRF2 signaling pathway has been attributed to the presence of an α,β-unsaturated carbonyl moiety [7]. Miranda-Sapla and co-workers [169] reported that *trans*-chalcone (**14**) modulates inflammatory response and enhances the total bound iron capacity via the activation of NRF2 and expression of HO-1 and ferritin. It also down-regulates ROS and NO levels in leishmania amazonensis-infected macrophages. Licochalcone A (**15**) induces nuclear translocation and activation of NRF2 through which it elevates the expression of the anti-inflammatory enzymes and determines licorice extract-induced lowered cutaneous oxidative stress in vivo [170]. Isoliquiritigen (ISL) (**16**), a natural chalcone compound, attenuates oxidative stress and inflammatory injuries via the activation of NRF2 signaling, as demonstrated in a mouse model of severe acute pancreatitis in which ISL determined a reduction in malondialdehyde, interleukin-6, tumor necrosis factor-α and cleaved-caspase-3 and an increase in NRF2, HO-1, NQO1 and superoxide dismutase (SOD) [171]. Chalcone flavokawain A (**17**) is a chalcone derivative that suppresses lipopolysaccharide-induced inflammation through activating the NRF2/ARE-mediated genes and inhibiting the ROS/NF-ĸB signaling in primary splenocytes [172].

### 4.5. Dimethyl Fumarate

Dimethyl fumarate (DMF) (**18**) (Table 1) is an α,β-unsaturated carboxylic acid ester, approved for the treatment of relapsing multiple sclerosis [8,12,173]. It exhibits significant antioxidant, anti-inflammatory and NRF2 activities due to the presence of α,β-unsaturated carbonyl moiety [111,174,175]. Akin et al. [175] reported that oral administration of DMF (**18**) alleviates oxidative stress via activation of NRF2/KEAP1 pathway in mouse ovary. Gopal et al. [176] reported evidence of NRF2 pathway activation in multiple sclerosis patients that were treated with DMF in Phase 3 studies. Ahuja et al. [177] observed that DMF (**18**) activates the NRF2 pathway, depletes glutathione level, decreases the viability of cells and inhibits mitochondrial oxygen consumption in a dose-dependent manner. Based on these observations, they recommended the development of monomethyl fumarate (MMF) a bioactive metabolite of DMF, which does not exhibit similar adverse effects, as a novel Parkinson’s disease drug [177]. In summary, the reactivity of α,β-unsaturated carbonyl system with thiols of the KEAPl cysteine residues is responsible for the activation of the NRF2 signaling pathway and accounts for the antioxidant and anti-inflammatory activities of α,β-unsaturated carbonyl-containing compounds. DMF is a notable multi-target compound that modulates NRF2, nuclear factor kappa-light-chain-enhancer of activated B cells (NF-ĸB), hydrocarboxylic acid receptor (HCAR2) pathways and regulates glutathione and iron metabolism which is utilized for the treatment of neurodegenerative diseases [178].

## 5. α,β-Unsaturated Sulfonyls

The sulfonyl group is an electron-withdrawing moiety found in several organosulfur compounds such as sulfones, sulfonamides and sulfonates [70,179]. The strong electron-withdrawing effect of the sulfonyl group accounts for the tendency of α,β-unsaturated sulfonyls to add to nucleophiles in order to form Michael-type adducts. This property also makes α,β-unsaturated sulfonyls to act as powerful dienophiles [180]. Several sulfonyl-containing compounds exhibit significant antioxidant and anti-inflammatory activities [181,182,183,184]. α,β-unsaturated sulfonyls are notable building blocks in the synthesis of organic compounds [185]. They exhibit notable biomedical significance [186]. They inhibit several enzymatic processes making them essential moieties in drug design and medicinal chemistry [89]. The first α,β-unsaturated sulfonyls were reported as potent inhibitors of cysteine proteases in 1995 [187]. They are inhibitors of cruzain, HIV-1 integrase, *Staphylococcus* aureus sortase, among others [188,189,190]. α,β-unsaturated sulfonyls reversibly inhibit diverse enzymes via conjugate addition reaction with the thiols of cysteine residue [187,190,191]. They are effective for intracellular inhibition of dipeptidyl peptidase1 [192,193]. α,β-unsaturated sulfonyls are reportedly activators of the NRF2 signaling pathway [2,9,23,32] as shown in Table 1.

### 5.1. Vinyl Sulfones

Vinyl sulfones (Table 1) have been reported as modulators of NRF2 activity due to the presence of the α,β-unsaturated sulfonyl system that accounts for their effectiveness as Michael acceptors [2,9,17]. Carlstrom et al. [2] reported that vinyl sulfone (**19**) activates the NRF2 signaling pathway with limited off-target effects on hypoxia-inducible factor 1 and NF-ĸB in PTRAF-transfected HEK293 cells. Lee and co-workers [23] also reported that compound **19** activates NRF2 signaling and induces the up-regulation of the expression of NRF2-dependent antioxidant enzymes in microglia. It inhibits the expression of proinflammatory enzymes and proinflammatory cytokines production in activated microglia. Woo et al. [32] reported that compound **19** in dopaminergic (DAergic) neuronal cells activates NRF2 and up-regulates the expression of NRF2-regulated antioxidant enzymes at mRNA and protein levels. It exerts neuroprotection and attenuates Parkinson’s disease (PD)-related deficits in PD mouse models [25]. Choi and co-workers [9,17] corroborated that compound **19** activates NRF2 and induces the expression of NRF2-regulated antioxidant mediators in PD mice. Although extensive researches have proven that compound **19** exhibits the highest NRF2 activity amongst its vinyl sulfone analogues, however, its poor drug-like properties remain a concern. In view of this, Choi et al. [17] designed a vinyl sulfone derivative (**20**) with improved NRF2 activation potency and drug-likeness. Compound **20** significantly induces NRF2 activation, up-regulation of NRF2-dependent genes, improves the movement ability in acute 1-methyl-4-phenyl-1,2,3,6-tetrahydropyridine-induced PD mice and reduces microglial activation and loss of DAergic neurons [17]. Vinyl sulfone derivative (**21**) is reportedly more potent than chalcone and vinyl sulfoxide analogues in activating the NRF2 signaling pathway and up-regulating the expression of HO-1 gene [32]. Vinyl sulfone compounds **22** and **23** induce the relief of H_2_O_2_-induced lesions, neutralize ROS, activate antioxidant response and promote neuroprotection via the activation of NRF2 pathway in PC12 cells. However, the neuroprotective activity of compound **22** is higher than that of compound **23** [194]. The electrophilicity and steric hindrance of α,β-unsaturated sulfones have been tuned to generate several potent NRF2 activators [194].

### 5.2. Vinyl Sulfonamides

Sulfonamides exhibit antioxidant and anti-inflammatory activities [195,196,197,198,199,200,201]. The presence of the α,β-unsaturated sulfonyl system in vinyl sulfonamides (Table 1) enable them to act as Michael acceptors and activate the NRF2 signaling pathway [17]. Choi and co-workers [17] synthesized several vinyl sulfonamides by substituting the sulfone moiety of compound **19** with sulfonamide moiety to improve NRF2 activation ability. The analysis of antioxidant enzymes and inflammatory cytokines expression in BV-2 microglial cells and SH-SY5Y human neuroblastoma cells, and of in vivo therapeutic effects on Parkinsonism in a mouse model of Parkinson’s disease showed that compounds **24**, **25**, **26**, **27**, **28** exhibit NRF2 activity and compound **26** is the most potent NRF2 activator. However, compound **26** is not as potent as the vinyl sulfonate analogues [17].

### 5.3. Vinyl Sulfonates

Sulfonates exhibit antioxidant and anti-inflammatory activities [202,203]. Vinyl sulfonate (Table 1) are highly activated Michael acceptors due to the α,β-unsaturated sulfonyl moiety they contain [17]. Vinyl sulfonate compounds **29**, **30** and **31** have been reported as potent activators of the NRF2 signaling pathway [17]. They exert therapeutic effects against Parkinson’s disease via their antioxidant, anti-inflammatory and neuroprotective activities [17]. Compound **29** exhibits about seven times NRF2 activity higher than its vinyl sulfone analogue (**19**). Compound **29** increases NRF2-related protein levels attenuates inflammation and decreases the production of NO in BV-2 cells. It also up-regulates the expression of NRF2-regulated antioxidant enzymes and inhibits motor deficits in Parkinson’s disease [17].

## 6. α,β-Unsaturated Sulfinyls

The sulfinyl group is available in several organosulfur compounds. It is a strong electron-withdrawing moiety and exhibits high configurational stability and several biological functions such as antioxidant, anti-inflammatory and NRF2 up-regulation activities [204,205,206,207]. Recently, sulfinyl group has been utilized in controlling the enantioselectivity of 1,4-additions involving carbon nucleophiles to α,β-unsaturated sulfoxides [208]. Similarly, α,β-unsaturated sulfinyl group is a very essential partner in Michael addition reaction involving thiols of the KEAP1 cysteine residues in NRF2 activation [209,210]. α,β-unsaturated sulfinyl compounds activate the NRF2 signaling pathway as shown in Table 1.

### Vinyl Sulfoxide

Sulfoxides exhibit antioxidant and anti-inflammatory activities [209,211]. The ability of vinyl sulfoxide (Table 1) to activate NRF2 and to induce HO-1 has been linked to the presence of an α,β-unsaturated sulfinyl system [32]. Woo and co-workers [32] synthesized vinyl sulfoxide (**32**) based on chalcone structure. In an attempt to determine the NRF2-activating potency of compound **32.** Woo et al. [32] assessed its ability to induce the expression of a NRF2-dependent genes in BV2 cells. Compound **32** was found to exhibit significant HO-1 inducing activity and confirmed to be a potent as its vinyl sulfone and chalcone analogues [32]. Shim et al. [3] designed and synthesized vinyl sulfoxide derivatives (**33** and **34**) using sulforaphane and gallic acid as structural templates and tested their HO-1 inducing ability as the measure of NRF2 activation in BV2 microglial cells. However, compounds **33** and **34** exhibit moderate HO-1 inducing activity and no inhibitory effect on NO production [3], thus suggesting that a more efficient electrophile is needed to get more effective NRF2 activator.

**Table 1:** α,β-Unsaturated moiety-bearing compounds as NRF2 activators/KEAP1 inhibitors.

S/N	Compound	Disease Studied	Model	NRF2 Activating Conc/Activity	Mechanism of Action	Biological Activity	Reference
8	**Sesquiterpene lactones** 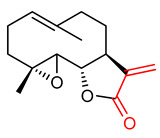 Parthenolide	Obesity	Mice, Adipocytes (3T3-L1), RAW264.7	1–8 µM	electrophilic modification of KEAP1 cysteine residues	NRF2 activation, Antioxidant, Anti-adipogenesis	[124]
		Obesity	3T3-L1 Cell	1–8 µM		NRF2 activation, Antioxidant, Anti-inflammatory	[125]
		Osteoporosis	Human	5–20 µM		NRF2 activation, Antioxidant, Anti-apoptosis	[126]
		Breast cancer	Human breast cancer cell line MDA-MB 231	2.0 µM		NRF2 regulation, chemoresistance	[133]
		Chronic lymphocytic leukemia	Human peripheral blood mononuclear cells (PBMCs)	1.46 µM		NRF2 activation, Antioxidant, cytotoxicity	[111]
9	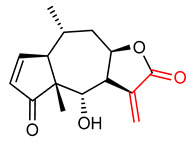 Helenalin	Acute hepatic injury	Male C57BL/6 Mice	0.75–3.00 mg/kg	electrophilic modification of KEAP1 cysteine residues	NRF2 activation, Antioxidant, Anti-inflammatory	[137]
10	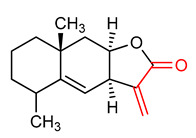 Alantolactone	Breast cancer	MCF-7 human breast cancer cells	10–30 µM	electrophilic modification of KEAP1 cysteine residues	NRF2 activation, anticancer	[142]
		Cancer	Heps1c1c7 cells	1–10 µM		NRF2 activation, Antioxidant, anticancer	[143]
		Chronic obstructive pulmonary disease (COPD)	Cigarette smoke-induced human bronchial epithelial cells	1–10 µM		NRF2 activation, Antioxidant, Anti-inflammatory	[144]
11	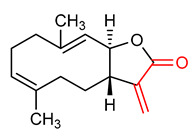 Costunolide	Acute liver injury	Mice	20–40 mg/kg	electrophilic modification of KEAP1 cysteine residues	NRF2 activation, Antioxidant, Anti-inflammatory	[146]
		Oxidative damage	PC12 Cells	5 µM		NRF2 activation, Antioxidant, neuroprotection	[150]
		Tumor	RAW264.7 Macrophages	0.1–1.0 µM		NRF2 activation, Anti-inflammatory	[147]
12	**Curcumin** 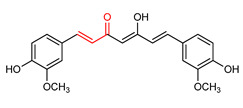	Neurodegenerative diseases	Neuronal cells	10 µM	electrophilic modification of KEAP1 cysteine residues	NRF2 activation, Antioxidant	[155]
		Oxidative stress, inflammation	HepG2 Cells	50 mg/kg		NRF2 activation, Antioxidant, Anti-inflammatory	[158]
		Nasal diseases	Human nasal fibroblast	0–5 µM		NRF2 activation, Antioxidant	[159]
		Oxidative stress	Rats	30 mg/kg		NRF2 activation, Antioxidant	[160]
13	**Prostaglandin** 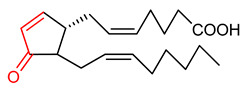 15-Deoxy-∆^12,14^-prostaglandin J2	Breast cancer	Human breast cancer cells	10 µmol/L	electrophilic modification of KEAP1 cysteine residues	NRF2 activation, Antioxidant	[163]
		Cancer	Mouse embryonic fibroblast (MEF) 293 cells	0.5–10 µM		NRF2 activation, Antioxidant, Anticancer	[164]
14	**Chalcones** 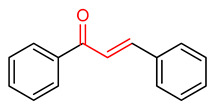 *Trans*-chalcone	Leishmannia amazonensis	L. amazonensis-infected macrophages	2–12 µM	electrophilic modification of KEAP1 cysteine residues	NRF2 activation, Antioxidant,	[169]
15	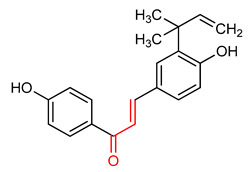 Licochalcone A	Cutaneous oxidative stress	UVA-irradiated human dermal fibroblast	9 µM	electrophilic modification of KEAP1 cysteine residues	NRF2 activation, Antioxidant, Anti-inflammatory	[170]
16	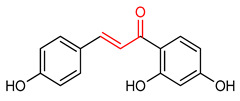 Isoliquiritigenin	Pancreatic injury	Mice	>3%	electrophilic modification of KEAP1 cysteine residues	NRF2 activation, Antioxidant, Anti-inflammatory	[171]
17	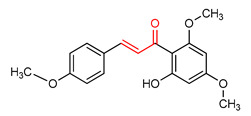 Chalcone flavokawain A	inflammation	Primary splenocytes	2–30 µM	electrophilic modification of KEAP1 cysteine residues	NRF2 activation, Antioxidant, Anti-inflammatory	[172]
18	**DMF** 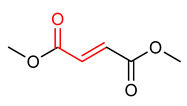 Dimethyl fumarate	Oxidative stress	Mouse ovary	20 mg/kg	electrophilic modification of KEAP1 cysteine residues	NRF2 activation, Antioxidant,	[175]
		Multiple sclerosis	Multiple sclerosis patient	0–400		NRF2 activation, Antioxidant,	[173]
		Parkinson’s disease	Mice	0.05–80 µM		NRF2 activation, Antioxidant, Anti-inflammatory	[177]
19	**Vinyl Sulfones** 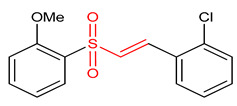 (E)-1-chloro-2-(2-((2-methoxyphenyl)sulfonyl)vinyl)benzene	Multiple sclerosis	HEK293	10 µM	electrophilic modification of KEAP1 cysteine residues	NRF2 activation, Antioxidant,	[2]
		Parkinson’s disease	PD animal model	1–20 µM		NRF2 activation, Antioxidant, Anti-inflammatory	[23]
		Parkinson’s disease	PD animal model	1–10 µM		NRF2 activation, Antioxidant, Neuroprotection	[32]
20	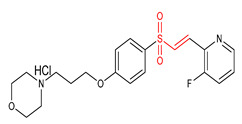 (E)-4-(3-(4-((2-(3-fluoropyridin-2-yl)vinyl)sulfonyl)phenoxy)propyl)morpholine hydrochloride	Parkinson’s disease	PD mice	0.3–10 µM	electrophilic modification of KEAP1 cysteine residues	NRF2 activation, Antioxidant, Neuroprotection	[9]
21	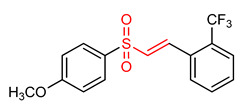 (E)-1-(2-((4-methoxyphenyl)sulfonyl)vinyl)2-(trifluoromethyl)benzene	Parkinson’s disease	PD mice	20 µM	electrophilic modification of KEAP1 cysteine residues	NRF2 activation, Antioxidant, Neuroprotection	[32]
22	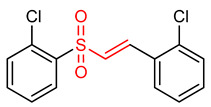 (E)-1-chloro-2-(2-((2-chlorophenyl)sulfonyl)vinyl)benzene	Oxidative stress	PC12 Cells	2.5–1.0 µM	electrophilic modification of KEAP1 cysteine residues	NRF2 activation, Antioxidant, Neuroprotection	[194]
23	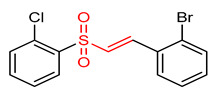 (E)-1-bromo-2-(2-((2-chlorophenyl)sulfonyl)vinyl)benzene	Oxidative stress	PC12 Cells	0.5–1.0 µM	electrophilic modification of KEAP1 cysteine residues	NRF2 activation, Antioxidant, Neuroprotection	[194]
24	**Vinyl Sulfonamides** 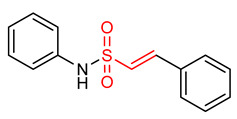 (E)-N,2-diphenylethenesulfonamide	Parkinson’s disease	PD mouse	>10 µM	electrophilic modification of KEAP1 cysteine residues	NRF2 activation, Antioxidant, Anti-inflammatory	[17]
25	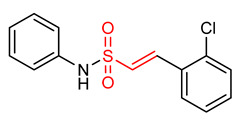 (E)-2-(2-chlorophenyl)-*N*-phenylethesulfonamide	Parkinson’s disease	PD mouse	>10 µM	electrophilic modification of KEAP1 cysteine residues	NRF2 activation, Antioxidant, Anti-inflammatory	[17]
26	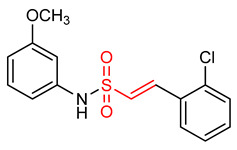 (E)-2-(2-chlorophenyl)-N-(2-methoxyphenyl)ethenesulfonamide	Parkinson’s disease	PD mouse	6.35 µM	electrophilic modification of KEAP1 cysteine residues	NRF2 activation, Antioxidant, Anti-inflammatory	[17]
27	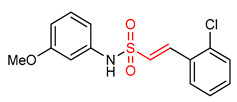 (E)-2-(2-chlorophenyl)-N-(3-methoxyphenyl)ethane sulfonamide	Parkinson’s disease	PD mouse	>10 µM	electrophilic modification of KEAP1 cysteine residues	NRF2 activation, Antioxidant, Anti-inflammatory	[17]
28	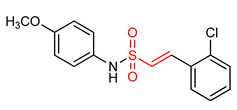 (E)-2-(2-chlorophenyl)-N-(4-methoxyphenyl)ethane sulfonamide	Parkinson’s disease	PD mouse	>10 µM	electrophilic modification of KEAP1 cysteine residues	NRF2 activation, Antioxidant, Anti-inflammatory	[17]
29	**Vinyl Sulfonates** 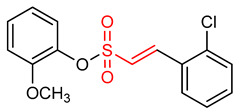 (E)-2-methoxyphenyl 2-(2-chlorophenyl)ethenesulfonate	Parkinson’s disease	PD mouse	0.076 µM	electrophilic modification of KEAP1 cysteine residues	NRF2 activation, Antioxidant, Anti-inflammatory	[17]
30	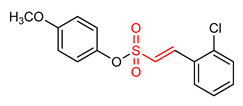 (E)-4-methoxyphenyl 2-(2-chlorophenyl)ethenesulfonate	Parkinson’s disease	PD animal model	0.237 µM	electrophilic modification of KEAP1 cysteine residues	NRF2 activation, Antioxidant, Anti-inflammatory	[17]
31	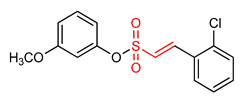 (E)-3-methoxyphenyl 2-(2-chlorophenyl)ethenesulfonate	Parkinson’s disease	PD mouse	0.165 µM	electrophilic modification of KEAP1 cysteine residues	NRF2 activation, Antioxidant, Anti-inflammatory	[17]
32	**Vinyl Sulfoxides** 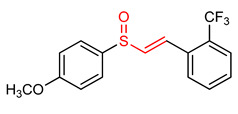 (E)-1-(2-((4-methoxyphenyl)sulfinyl)vinyl)-2-(trifluoromethyl)benzene	Parkinson’s disease	BV-2 Cells	20 µM	electrophilic modification of KEAP1 cysteine residues	NRF2 activation, Neuroprotection	[32]
33	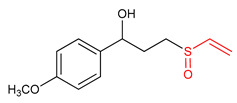 1-(4-methoxyphenyl)-3-(vinylsulfinyl)propan-1-ol	Parkinson’s disease	BV-2 Cells	20 µM	electrophilic modification of KEAP1 cysteine residues	NRF2 activation, Antioxidant,	[3]
34	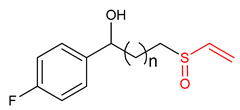 1-(4-fluorophenyl)-3-(vinylsulfinyl)propan-1-ol	Parkinson’s disease	BV-2 Cells	20 µM	electrophilic modification of KEAP1 cysteine residues	NRF2 activation, Antioxidant,	[3]

**Table 2:** α,β-unsaturated moiety-bearing NRF2 activators in clinical trial or approved by FDA.

Entry	Compound	Clinical Trial/FDA Approval	Targeted Disease	Reference
8	Parthenolide	Clinical trial	Cancer	NCT00133341
12	Curcumin	Clinical trial	Impaired glucose tolerance and insulin resistance/ type 2 diabetes	NCT01052025
15	Licochalcone A	Clinical trial	Human oral squamous cell carcinoma	NCT03292822
18	Dimethyl fumarate	FDA approved	Multiple sclerosis	NCT00810836

## 7. Pharmacological Profile of α,β-Unsaturated Structure-Bearing NRF2 Activators

The α,β-unsaturated moiety-bearing compounds activate the NRF2/KEAP1 signaling pathway significantly. Generally, these compounds have moderate to good lipophilicity, oral bioavailability, pharmacokinetic, pharmacodynamics and toxicological profile. α,β-unsaturated moiety-bearing molecules such as parthenolide, helenalin, alantolactone, prostaglandins, vinyl sulfonamides and sulfoxides permeate the blood–brain barrier in a dose-dependent manner and therefore serve as potential therapeutic agents for CNS-related diseases. Micro- and nano-formulation of α,β-unsaturated carbonyls such as prostaglandins and curcumin improve their pharmacological profile. α,β-unsaturated-structure bearing sesquiterpene lactones obtained from feverfew plant have lipophilic character which generally affects their potency [212,213]. For instance, parthenolide (**8**) exhibits a significant lipophilicity which may explain its good blood–brain barrier permeability and cytosol penetration for KEAP1-NRF2 signaling pathway activation [214]. Therefore, it has low solubility in water with reduced bioavailability, which has limited its potential clinical application as an anticancer drug molecule [114]. However, the pharmacokinetics, pharmacodynamics and bioavailability of parthenolide (**8**) have been improved in its derivatives such as dimethylaminoparthenolide (DMAPT) and others which exhibit improved oral bioavailability and ADME properties but display similar mechanism of action to parthenolide (**8**) [215,216]. Helenalin (**9**) is a lipophilic compound which penetrates cell membranes and exhibits high cytotoxicity. It has low oral bioavailability in vivo and considerable lipophilicity which can be modified [136,217]. Helenalin is toxic but it is considered generally safe when applied topically to humans. The oral LD50 of helenalin (**9**) has been obtained as 85–150 mg/kg [218,219]. Oral administration of helenalin (**9**) exhibits higher toxic effect than parenteral administration [220,221]. In addition, the specificity, pharmacokinetics and metabolism of helenalin (**9**) should be further investigated in the light of NRF2 activation. Alantolactone (**10**) is lipophilic and permeates the blood–brain barrier, making it a NRF2 activator that can be explored for CNS-related diseases in which oxidative stress and inflammation have been implicated [222,223]. The pharmacokinetics and metabolism of alantolactone (**10**) has been widely reported [224,225,226]. It has been observed that after oral and intravenous administration, alantolactone (**10**) displays low toxicity, absorption and rapid elimination. The metabolism of alantolactone involves its conjugation with thiols in which the α,β-unsaturated carbonyl moiety is preferred as the structural metabolic site. This feature enhances its activation of the NRF2 signaling pathway. It exhibits low oral bioavailability due to its low aqueous solubility [224,225,226]. Costunolide (**11**) is lipophilic with low polarity, water solubility and good storability [227,228]. The pharmacokinetic assessment of costunolide (**11**) was reported by Zhang and co-workers [229]. It takes 10.46h for costunolide to reach the maximum plasma concentration (Tmax) of 1.29µg/mL and its elimination half-life (t_1/2_) is 5.54 h. The pharmacokinetics’ area under the curve (AUC) of costunolide (**11**) is 308.83 ngh/mL which represents the area under the graph of blood plasma concentration against time after the oral administration of a dose. It describes the actual body exposure to costunolide (**11**) after dosage [229]. Costunolide (**11**) has a higher bioavailability and lower clearance and volume of distribution than several sesquiterpene lactones including dehydrocostus lactone [230].

Curcumin (**12**) is highly lipophilic, with low water solubility (11ng/mL), poor absorption, bioavailability and rapid metabolism. These features have limited its effectiveness and usefulness as drug molecule [231]. The maximum curcumin level in patients is 1.8–11 nM even when high doses of curcumin (**12**) is administered per day [232]. It has a short half-life (t_1/2_) of <45 min and <30 min for oral and intravenous administration. However, the problem of poor absorption and low bioavailability of this α,β-unsaturated carbonyl-based flavonoid polyphenol has been solved by the discovery of micro- and nano-formulated curcumin with >100-fold enhanced absorption and high bioavailability [233]. This requires evaluation of the NRF2 activity of these formulated curcumins.

Prostaglandins exhibit high lipophilicity and permeate cells through prostaglandin transporters. They exert their pharmacological effect via binding to prostaglandin receptors [234]. They are administered topically, orally, intravenously and by inhalation [235,236,237]. Their toxicity is therapy-dependent; however, they are tolerated when it is limited [238]. 15-Deoxy-∆^12,14^-prostaglandin J_2_ (15d-PGJ2) (**13**) when administered at high doses, stimulate anti-inflammatory and anti-proliferative dual actions [239]. 15d-PGJ2 (**13**) exhibits biphasic pharmacodynamics, and this imposes some difficulties when it is used in free form [240]. Again, using it at low dose or in an uncontrolled manner induces a reverse response that could worsen a disease condition [239]. Cell proliferation and apoptosis are induced by 15d-PGJ2 (**13**) at low and high doses respectively [240]. Due to its lipophilic nature, 15d-PGJ2 (**13**) finds it difficult to penetrate the aqueous cytosol at low dose; therefore, a high dose of this compound is required for an effective activation of the cytosolic KEAP1-NRF2 signaling pathway [239]. In an attempt to improve the solubility, pharmacokinetics and tissue targeting of 15d-PGJ2 (**13**), its nano-formulations such as poly (D,L-lactide-coglycolide) (PLGA) nanocapsules, albumin conjugates and liposomes have been developed [241,242,243].

Chalcones are lipophilic in nature and the linker fragment, an α,β-unsaturated carbonyl system is the main pharmacophore required for NRF2 activation [24,244]. The pharmacokinetic evaluation of chalcones shows that several chalcone analogues have low bioavailability, distribution, rapid metabolism and elimination [245]. The LD50 of *trans*-chalcone (**14**) in mouse was found to be 56 mg/kg when administered intravenously and >500 mg/kg when it was administered orally and intraperitoneally, this affects its toxicity [246,247,248]. Licochalcone A (**15**) exhibits poor absorption and bioavailability (3.3%). It displays plasma concentration level in the range of 0.53–530 ng/mL in rat and AUC of 2479.9 and 243.3 ngh/mL for intravenous and oral administration [249]. Isoliquiritigenin (**16**) shows absorption percentage of 10.36%, AUC of 0.67 µgh/mL, poor solubility, low bioavailability and rapid elimination at 35 mg/kg oral administration in mice [250]. Chalcone flavokawain A (**17**) exhibited AUC of 18.0 mgh/mL, Cmax value of 0.7mg/L, Tmax value of 0.942 h and half-life of 2.021 h in mice after oral administration [251].

The pharmacological profile of DMF (**18**) as α,β-unsaturated carbonyl-bearing NRF2 activator has been evaluated, and the USA Food and Drug Administration (FDA) has approved it for the treatment of multiple sclerosis [12]. However, its side effect of 30% decrease in the lymphocyte count after administration remains a challenge [12,252].

Vinyl sulfones have low solubility in water [253]. Methyl vinyl sulfones have LD50 of 570 mg/kg and 32 uL/kg based on oral and skin administration respectively in rats and rabbits [254]. Vinyl sulfone analogues reportedly displayed desirable pharmacokinetic and safety profile in animals such as dogs, primates and rodents [255,256]. Compound **19** shows poor metabolic stability, solubility and cytochrome P (CYP) inhibition. It displays poor safety as it blocks >50% of CYP activity after treatment with 10 µM dose. It is metabolically unstable as only 20% of it remains after incubation for 30 min with the microsomes of human liver [9]. Compound **20** displayed excellent plasma stability in humans and rats: 98.2 and 90.2% of it remained after incubation for 30 min. It permeates the blood–brain barrier and exhibits favorable CNS drug permeability of 15.6 × 10^−6^ [9]. Pharmacokinetically, compound **20** shows rapid absorption, maximum concentration time of 0.4 h after dosage and oral bioavailability of 45.3% in rats [9]. Compounds **21**, **22** and **23** can permeate the neuronal cells and activate NRF2 but their pharmacological profiles need to be further evaluated [25]. Sulfonamides are lipophilic and their vinyl analogues permeate the cytosol to activate the KEAP1-NRF2 signaling [17,257]. The pharmacokinetic and pharmacodynamics properties of sulfonamides have been widely reported [258,259]. However, these properties need to be determined for vinyl sulfonamides (**24**–**28**). Sulfonates, sulfoxides and their vinyl analogues (**29**–**34**) are lipophilic and permeate cytosol but the pharmacokinetic and pharmacodynamic properties of their vinyl analogues are yet to be reported [17,32,260].

## 8. Structure–Activity Relationship of α,β-Unsaturated Structure-Bearing NRF2 Activators

**Scheme 5:**
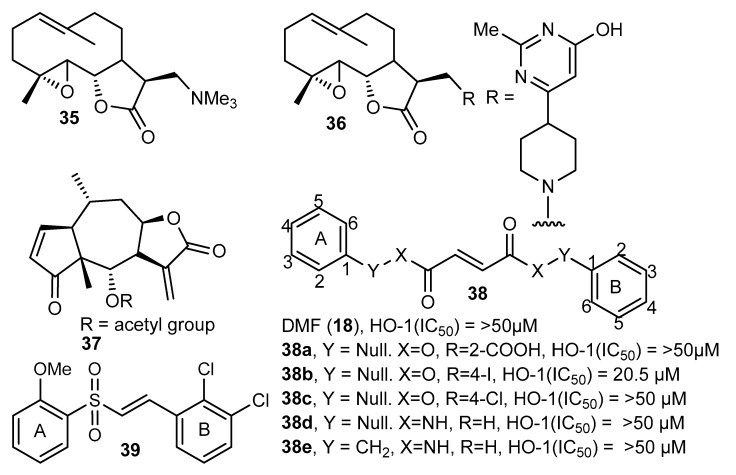
Structure–activity relationship of α,β-unsaturated moiety-bearing compounds.

The bioactivities of NRF2-activating sesquiterpene lactones are mainly dependent on their α-methylene-γ-butyrolactone (αMγB) structural composition [261,262]. The αMγB contains the α,β-unsaturated carbonyl system, which reacts with cysteine for NRF2 activation [262,263]. In parthenolide (**8**), the structural replacement of the ethylene group of α,β-unsaturated carbonyl of the αMγB with dimethylamino group that results in the formation of DMAPT (**35**) (Scheme 5) improves the pharmacological profile and induces NRF2 nuclear localization [215,261]. The replacement of the ethylene group with 2-methyl-6-(1-methyl-piperidin-4-yl) pyrimidin-4-ol in compound **36** (Scheme 5) reportedly determined a better biological activity, ADME property and safety profile when compared to parthenolide (**8**) and DMAPT (**35**) [264] (Scheme 5). However, an improved NRF2 activity has not been reported about compound **36**. In helenalin (**9**), the presence of OH group decreases its lipophilicity but the modification of the α-methyl-γ-lactone containing the α,β-unsaturated carbonyl moiety alters the lipophilicity and improves the pharmacological properties of helenalin (**9**) [136,217]. The substitution of H with acetyl group in OH group of helenalin derivative (**37**) (Scheme 5) increases the toxicity of helenalin (**9**) [265]. The incorporation of amine into the αMγB structure of alantolactone (**10**) and costunolide (**11**) enhances their aqueous solubility and selective binding as Michael acceptors which may affect their activation of the NRF2 signaling pathway [262,266].

The α,β-unsaturated carbonyl structural system of chalcones elicits its NRF2-activating effect [7]. The incorporation of CF_3_ into ring B of chalcone (**14**) improves its NRF2 activation, and the ortho CF_3_-substituted derivative has been found to be non-cytotoxic and to exhibit the highest activity. Conversely, the ortho substitution with −NO_2_ increases toxicity and decreases NRF2 activation [267]. The incorporation of 3,4-dihydroxyl group into ring A of compound **14**, improves the neuroprotective activity of chalcone via free radical scavenging and NRF2 activation, in contrast to what is observed with the introduction of the same 3,4-dihydroxyl group into ring B [268].

The α,β-unsaturated dicarbonyl structural system of DMF (**18**) is the central chain essentially responsible for HO-1 induction and NRF2 activation [269]. The addition of phenyl rings directly to the carboxylic groups of DMF (**38**) results in comparable or better HO-1 inducing activity than DMF (**18**). The addition of 2-COOH, 4-I and 4-Cl to the two phenyl rings (**38a**–**e**) improves the potency of these DMF derivatives (**38a**–**c**) as HO-1 inducers. The substitution of the ester group with an amide residue (**38d**,**e**) significantly enhances their HO-1 induction and directly improves their NRF2 activation [269] (Scheme 5).

The α,β-unsaturated sulfonyl structural system determines the NRF2-activating effect of vinyl sulfones, sulfonamides and sulfonates (**19**–**31**) [9,17]. The incorporation of Cl− group to the ortho position of ring B (**39**) (Scheme 5) improves the NRF2 activation of compound **19** while substitution with *o*-pyridine and F− decreases the NRF2 activation. The methoxy group at position 2,3 and 4 of ring A was found to increase the NRF2 activation of compound **19** with 4-OMe substitution being the highest [9]. OMe− and Cl− at position 2 of ring A and B respectively in vinyl sulfonamides (**24**–**28**) resulted in improved NRF2 activation [17]. Similarly, the addition of OMe− and Cl− at position 2 of ring A and B respectively in vinyl sulfonates (**29**–**31**) elicits the highest NRF2 activation [17]. The introduction of OMe−, F− and OH− groups to α,β-unsaturated sulfinyl-bearing sulfoxides (**33**–**34**) improves the HO-1 induction and subsequently the NRF2 activation [3] (Scheme 5). Taken together, for sesquiterpenes lactones, the presence of −NMe_3_ in the αMγB moiety increases the NRF2 activity of parthenolide while the presence of –NH_2_ enhances the NRF2 activity of alantolactone and costunolide. Generally, the presence of CF_3_ in the ring B of chalcones improve their ability to activate the NRF2 signaling pathway. Another approach to increase the NRF2 activity of α,β-unsaturated bearing compounds is to incorporate another α,β-unsaturated moiety which entails the presence of double Michael acceptors that will form conjugates with thiol groups of the KEAP1 cysteine residues. This type of structural modification gives such compounds advantage over their monofunctional analogs in terms of NRF2 activity as in compounds such as curcumin and triterpenoids. Based on the SAR studies, structural modification of α,β-unsaturated moiety-bearing compounds enhances their NRF2 activity, limits the off-target effects common to several electrophilic NRF2 activator and improves their ability to permeate the blood–brain barrier, a therapeutic tool in neurological diseases.

## 9. α,β-Unsaturated-Based NRF2 Activator in Parkinson’s Disease

Taken together, the performance of these α,β-unsaturated moiety-bearing NRF2 inducers in Parkinson’s disease is worthy of attention. Parkinson’s disease can be described as a neurodegenerative disease characterized by loss of balance, rigidity, postural instability, slow movements and tremors. It is well established that pathophysiologically, this disease sets in due to gradual loss of cells in the dopamine-producing area of the brain known as substantia nigra which occasions deficiency of dopamine that results in weakened muscle activities, loss of balance and movement disorder. A large body of evidence indicates that oxidative stress has been implicated in Parkinson’s disease and NRF2 being a key regulator of endogenous antioxidant has been found a worthy therapeutic target in the disease. Interestingly, the evidence discussed thus far shows that α,β-unsaturated moiety-bearing compounds are the most studied NRF2 activators in Parkinson’s disease due to their therapeutic potentials. About 60% of the α,β-unsaturated-based NRF2 activators reported were targeted against Parkinson’s disease (Table 1). It shows that compounds such as DMF, vinyl sulfones, vinyl sulfonamides, vinyl sulfonates and vinyl sulfoxides are drug candidates for Parkinson’s disease. The available data indicate that the preference for compounds containing α,β-unsaturated moiety in Parkinson’s disease treatment is related to their ability to attenuate ROS-mediated dopamine neuronal damage via NRF2 activation.

## 10. Conclusions

This update on the NRF2 activity of α,β-unsaturated moiety-bearing compounds shows that these compounds are essential for the control of pathological mechanisms of diseases in which oxidative stress has been implicated. Several pharmacological activators of the NRF2 signaling pathway are electrophilic molecules, most of which are compounds bearing at least an α,β-unsaturated structure. α,β-unsaturated moieties are abundant in natural and synthetic compounds. α,β-unsaturated moiety of carbonyl, sulfonyl and sulfinyl groups are the most reported, probably due to their relatively strong electron-withdrawing effects. In comparison to the α,β-unsaturated carbonyl group, limited work has been published on the role of α,β-unsaturated sulfonyl and sulfinyl moieties in NRF2 activations, probably due to their scarcity in nature and limitations in synthesis. Moreso, α,β-unsaturated carbonyl-based compounds are the most effective NRF2 activator due to their high reactivity, ease of covalent ligand binding to target proteins, thiol trapping and ability to react with a wide range of cys-bearing amino acids, peptides and proteins. The mechanism of action of these α,β-unsaturated systems may vary slightly, but ultimately leads to the same disruption of the KEAP1-NRF2 complex, electrophilic modification of KEAP1 cysteine residues and activation of the NRF2 signaling pathway. The reactivity of α,β-unsaturated systems in Michael addition reactions is influenced by substitution pattern because the nature and position of substituents in compounds containing these moieties affect their chemical reactivity with thiols and hence their biological activities. The current review provides useful information for researchers to evaluate more α,β-unsaturated-based compounds for NRF2 activity in order to identify lead compounds for the development of potent and novel NRF2 activators in the treatment of diseases caused by oxidative stress. However, future research should be directed towards improving their pharmacological properties via structural modification and harnessing them for specific diseases such as Parkinson’s disease. Some of the compounds that have performed well as NRF2 activators should be subjected to clinical trials.

## Data Availability

Not applicable.
